# Understanding the role of stress, personality and coping on learning motivation and mental health in university students during a pandemic

**DOI:** 10.1186/s40359-022-00971-w

**Published:** 2022-11-10

**Authors:** Chris Gibbons

**Affiliations:** School of Psychology, Queen’s University Belfast, Belfast, BT7 1NN UK

**Keywords:** Stress, Eustress, Pandemic-related stress, Control, Support, Personality, Mental health, Learning motivation

## Abstract

The aims explored the associations between stress, personality and coping on student mental health and compared defensive-pessimism and optimism as influences on learning motivation. Most research construes ‘stress’ as ‘distress’, with little attempt to measure the stress that enhances motivation and wellbeing. Undergraduate psychology students (N = 162) were surveyed on student and pandemic-related stressors, personality, support, control, mental health and learning motivation. Overall, adverse mental health was high and the lack of motivation acute. While positive ratings of teaching and optimistic thinking were associated with good mental health, context control was key. Adverse ratings of teaching quality lowered learning motivation. Support and conscientiousness bolstered learning motivation and conscientiousness buffered against the adverse impact of stress on motivation. Openness was associated with the stress involved in learning. For those anxious-prone, defensive-pessimism was as effective as optimism was in stimulating learning motivation. Developing context control, support and strategies linked to personality could bolster student resilience during and post Covid-19.

## Introduction

Stress has historically been defined as a physiological and psychological response [1, 2], and as the external stimuli that trigger or result in that reaction [3]. This early stimulus–response framework saw psychological factors as largely a *consequence* of the stress response. In contrast, in the Transactional model of stress, psychological and social factors are front and centre in recognizing and interpreting demands (the primary appraisal) and in managing those demands (the secondary appraisal). Adopting this model, stress is defined as the demands that exceed one’s capacity to cope [4].

**Fig. 1:**
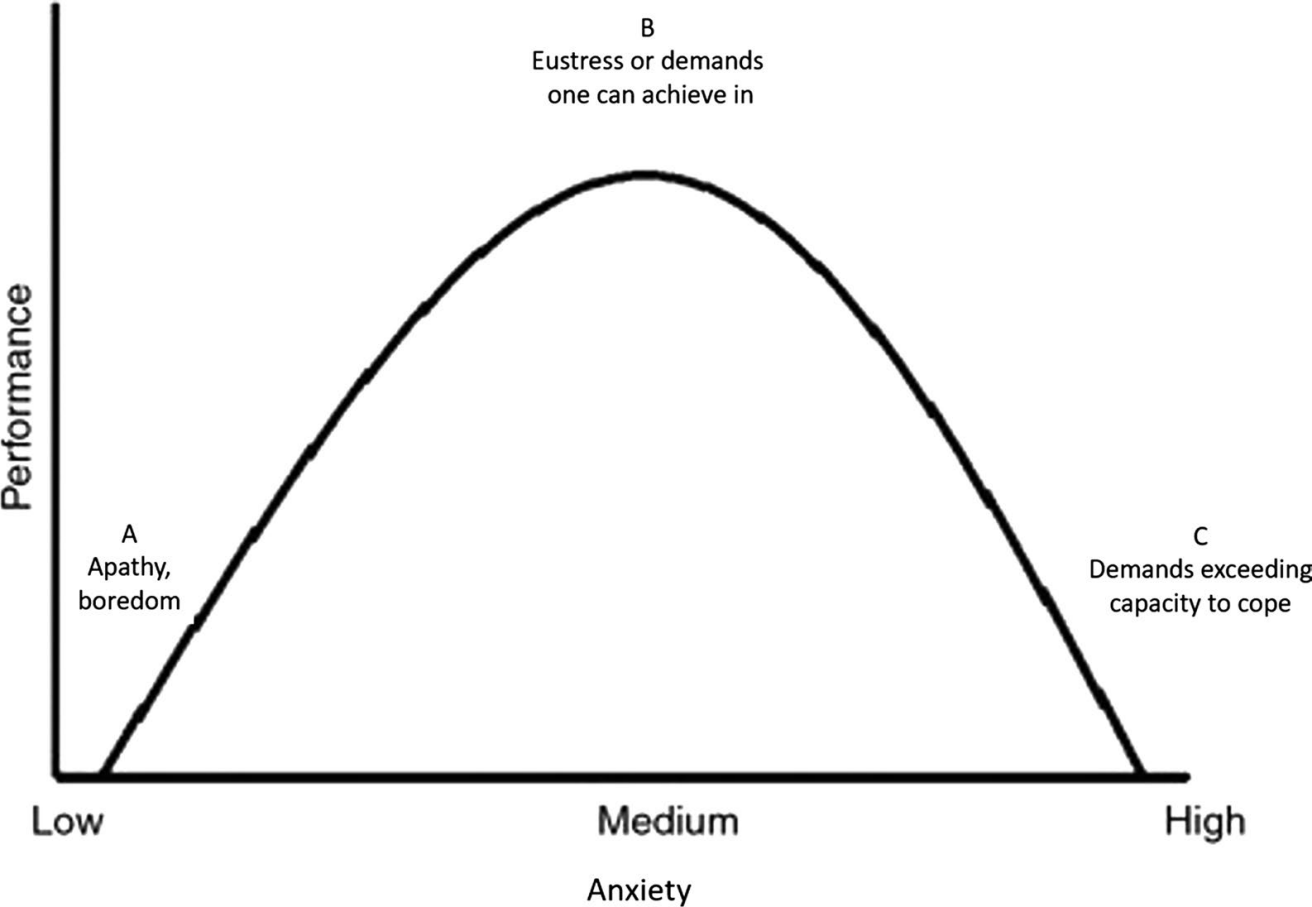
Adapted from the Yerkes–Dodson curve (1908) [7]

The primary appraisal refers to the initial perception and assessment of the stressor. This can lead to the judgment that it is irrelevant (or benign), a challenge or a threat. As illustrated in Fig. 1, sources of stress that are interpreted as demands in which one can achieve are called eustress (B) and those that are perceived as associated with apathy or boredom (A) or, more often, as exceeding one’s capacity to cope (C), are sources of distress [5]. The traditional health psychology approach construed stress in terms of degrees of distress. This study adopted a positive psychology perspective with university demands measured using an adapted National Student Survey (Higher Education Funding Council in England, 2017), with a response scale that allowed stress demands to be rated as hassles (that hold the potential to have an adverse effect on wellbeing) *and* as uplifts (that hold the potential to enhance wellbeing). This is consistent with the ‘threat’ and ‘challenge’ or distress and eustress primary appraisal judgments in the Transactional model. This study measured daily and ongoing demands, rather than life-events. This is consistent with Moos and Swindle’s (1990) argument that daily and ongoing stressors are important influences on wellbeing [6].

## Sources of student stress

Sources of student stress include academic demands, such as coursework, assessment, exams and work-life balance [8–10]; to fear of failure and lack of timely feedback on assessments and to the quality of teaching [11–13]. Personal sources of stress include financial concerns, managing apparent free time, frequently working part-time while studying, and concerns about future careers [13]. The changes students experience as they transition to university are frequently a source of acute stress. For most, they are learning to live independently, meet new people and often live in close confines with strangers, as well as managing their own finances, and all along with the challenges posed by a course that may leave them feeling overwhelmed [14].

## Stress effects in students

Wellbeing is defined as: ‘…a state of complete physical, mental and social wellbeing and not merely the absence of disease and infirmity’ [15]. While critics question the assumption of ‘completeness’ as integral to wellbeing, the definition highlights the critical role of psychology in wellbeing. Perceived stress can affect student wellbeing, including depression [16]; happiness [14] and even suicidal ideation [17]. Macaskill (2012) reports that students under 26 report more adverse wellbeing because they are still transitioning into adulthood [18].

A widely used measure of self-reported mental health is the General Health Questionnaire (GHQ), with approximately, 15–19% of the general population categorised as ‘at risk’ of developing a stress-related illness based on this measure [19]. This is not a life-threatening illness but complaints ranging from tension headaches, back problems, mouth ulcers and cold sores to digestive and intestinal problems, mood swings and irritability. Among student populations, this can range from 30% to over 60% [11, 20]. These stress effects have been observed in students in the UK; in North America [21, 22]; Australia [23] and Sweden [24] and the experience of stress has been directly linked to student attrition and retention issues [25].

## Pandemic stressors and effects

Following the global spread of the Covid-19 virus, a UK national lockdown was declared on 23^rd^ March 2020. This led to a dramatic change in students’ university experience. Learning and teaching became a virtual experience, with students, in this sample, receiving online pre-recorded lectures, live virtual seminars and tutorials. Rogowska, Kusnierz and Bokszczanin (2020) examined stress, coping and wellbeing in Polish students (n = 914) during Covid lockdown. Self-rated health and anxiety were poorer compared to normative data and those high in perceived stress more frequently used emotion-based coping [26]. Awoke, Mamo, Abdu and Terefe (2021), reported that over a third of health-professional students (n = 337) in Ethiopia, surveyed during the pandemic, reported high perceived stress [27]. However, neither study was longitudinal. Elmer, Mepham and Stadfeld (2020) measured the sources of stress and wellbeing in students (n = 212) before and after the onset of the pandemic in Sweden [28]. Within sample comparisons showed marked increases in depression, anxiety, loneliness and distress. Key sources of stress included the health of family and friends and uncertainty about their future, along with physically and emotionally isolation.

In a survey of 69,054 quarantined students in France, between April and May 2020, Wathelet et al., (2020) found that students worried more about any symptom of illness, indicating high anticipatory anxiety and the loss in part-time income was associated with higher anxiety, depression and even suicidal ideation [29]. In a longitudinal survey of 454 students in Italy, higher rates of mental health symptoms, related to depression, anxiety and obsessive–compulsive tendencies, were reported during lockdown, compared to when restrictions were lifted and females suffered disproportionately more [30]. A similar sex difference and overall deterioration in physical and mental health was observed in a longitudinal test in May and June, 2020, in university students in Germany (n = 917), [31].

What seems to add to the weight of stress and mental health concerns is not just the impact Covid might have on students but on their family and friends too. Of 7,143 students surveyed in China in January and February 2020, those whose family and friends had Covid, scored higher on anxiety [32], and similar results were found among students in Spain [33].

Since October 2020, the Office for National Statistics in England (ONS) have carried out three pilot surveys of university students (in mid-October and the start and end of November) with over 100 000 students in England and Scotland invited to participate via emails from the National Union of Students [34]. Between 2016- to pre-pandemic 2020, wellbeing measures (operationalised through life satisfaction, life worthwhile, happiness and anxiety), had already declined for students compared with similar aged non-students in the general population [35]. The differences are likely to be influenced not just by the increased demands and life changes students faced but that students are typically more willing, than non-students, to share mental health issues and university cultures are more supportive and focused on addressing student mental health.

In the Student Academic Experience Survey, a UK wide UCAS survey of first year students, taken in March, after most students had stopped face-to-face teaching, there was a drop in happiness scores [35]. The results from these and other large surveys [34, 36] lays testament to the adverse impact Covid-19 has had on students’ lives and mental health.

## Coping with stress

The secondary appraisal refers to individual coping resources, personality and the past experiences drawn on to perceive and manage stress demands. Key student coping resources include support [37] and control [13, 38, 39]. While trait-related control is a strong predictor of good coping, so is context control or the skills one acquires to feel in control in a given situation [11, 40, 41]. Given the potential context control has over trait-related control in improving coping, it is this type that is measured. Important personality ingredients, related to coping, include those measured by the Big Five [42], including extraversion [43] and conscientiousness, levels of emotional stability and openness [44]—in education contexts, openness is important if learning is to expand; and optimistic thinking strategies have been associated with improved wellbeing, performance and health [45–47]. Those scoring high on optimism construe stress demands in a way that makes success more likely. They tend to perceive change and stress demands as opportunities to grow and achieve, for example, those who cope well more frequently score stress demands as higher on uplifts and lower when rated as hassles [12]. They are biased to attend more to positive events over negative events (called defensive optimism) and they are more active in learning from their coping mistakes [4].

Norem and Cantor (1986) dispute the claim that adopting optimistic thinking strategies offers a panacea to the downside of stress [48]. They argue that for those anxious-prone, a more effective strategy is defensive pessimism. This involves setting yourself unrealistically low expectations in situations that cause you anxiety. Setting a high expectation of success could add to already heightened anxiety and inhibit performance, tipping you past the peak of the curve in Fig. 1.

## Aims and hypotheses

Most of the research into student stress and coping comes from pre-pandemic findings and the pandemic forced universities to turbo-charge their digital learning provision, providing a different environment to explore the role of stress and coping on mental health and learning motivation. Despite the education potential that digital and remote learning holds, its impact on learning motivation in higher education is mixed [49–51]. This underscores the need to explore its effect on student motivation during a pandemic, along with the coping and moderating influence played by personality, support and control on motivation and mental health. The factors affecting the rating of stress associated with achievement (i.e., eustress) is a relatively under-researched area in students [13, 41]. This study, therefore, aims to explore the relationship between sources of stress (rated as hassles and again as uplifting opportunities) and influences on coping (ratings on support, context control and personality) on mental health and learning motivation. A second aim was to see if defensive pessimism, compared to optimism, was an effective strategy to harness anxiety as motivation towards learning goals.

The following hypotheses were tested:

- **H1:** There will be a difference in the mental health of students studying during the pandemic compared to pre-pandemic norms.
- **H2:** There will be a difference in stress ratings (on hassles and uplifting ratings and pandemic-related stress) between those ‘at risk’ and ‘not at risk’ of a stress-related illness.
- **H3:** There will be correlations between sources of stress, support, control and personality and the outcomes – mental health and learning motivation.
- **H4:** Support, control and personality will have a moderating influence on the impact stress has on mental health and learning motivation.
- **H5:** There will be no difference in learning motivation between those high on defensive pessimism and optimism.

## Methods

### Design

A survey-based, correlational design was employed. The predictor variables were: course-related demands (rated as hassles and as uplifts), amended from the National Student Survey; pandemic-related stressors, including social media use and changes in diet and exercise; and, finally, aspects and influences on coping, namely support, context control and personality.

### Participants

A sample of 162 university students (81% of the second-year cohort) were recruited from the second-year of a psychology BSc programme. The inclusion criteria were second year full-time psychology students. Part-time students and those first year were excluded to avoid conflating the different, additional demands they face with those measured (e.g. related to time management and transitioning to university). On demographics, 86.4% were female (n = 140) and 13% male (n = 21). Participants’ average age was 22 years (SD = 4.55 and range 18–59 years).

### Materials

Students completed an online survey that included a brief and instructions and 89 items gathering information on demographics, sources of student stress, influences on coping—control, support and personality and on anxiety, course satisfaction, learning motivation and mental health.

### Procedure

The cohort was made aware of the study via email and in links on their course homepage to a google survey link. Participation was voluntary and respondents were told they could stop at any time without penalty. The survey took approximately 12 min to complete. They were given the opportunity to complete this in class.

### Measures

#### The national student survey (NSS) [52]

NSS items were adapted so participants could rate each item twice – once as a “hassle” (a perceived source of distress) and once as an “uplift” (a perceived source of eustress). A continuous response scale, from 0 to 5, was used to rate each item as a hassle or uplift – 0 indicating that the item caused no source of distress or eustress and 5 indicating an extreme source. A range of factors were measured using 23 items from the NSS, such as teaching demands, assessment and feedback, time management etc. An example item is: ‘The extent to which teaching staff explain things’. Banked items from the NSS were selected to measure *learning motivation*. This was a two-item measure with a 5-point Likert scale. An example item is: ‘I have found the course motivating’. The Alpha coefficients for all factors ranged from .64–.85.

#### Pandemic-related stressors (generated by the author)

This scale contained six items that split into two sub-scales: *time on devices* and *lack of motivation*. They were generated following focus group interviews with three groups of second year students. Respondents rated each item on a 10-point response scale from 1 (Not at all True) to 10 (Very True). Sample items included: ‘During the period of Covid-19 restrictions, have you found that you have been: ‘…using social media more than usual’ (time on devices), ‘…losing your mojo’ (lack of motivation) The Alpha coefficient for *time on devices* was .67 and *lack of motivation* .85.

#### Context control [12]

This scale, of three items, aimed to measure how much participants had developed control in specific contexts. A 5-point Likert scale was used. A sample item is: ‘The pace of learning often leaves me with little feeling of control.’ Two of the three items are reverse scored. The Alpha coefficient was .80.

#### The values in action scale [53]

This eight-item scale measures levels of optimistic thinking. Participants respond on a five-point Likert scale. A sample item is: ‘I always look on the bright side’. The Alpha coefficient was .81.

#### Big five inventory-10 (BFI-10) [54]

This is a ten-item scale using a 5-point Likert scale. Respondents are asked to rate statements that describe their personality. A sample item is: ‘I see myself as someone who is reserved’. Two items measure each of the Big Five traits, with one of those two being reversed. Alpha coefficient ranged from .61–.74.

#### Defensive pessimism scale [48]

This is a twelve-item scale using a 7-point response scale from ‘Not at all true of me’ (1) to ‘Very true of me’ (7). A sample item is: ‘I often start out expecting the worst, even though I will probably do okay’. The Cronbach’s alpha was .87.

#### General health questionnaire (GHQ) [55]

This a twelve-item scale and respondents answer on a four-point frequency scale. GHQ measures general levels of self-confidence, happiness, anxiety, depression and sleep disturbance and, taken together, this comprises a general measure of mental health. An example item is: ‘Have you recently been able to concentrate on whatever you’re doing?’ Response options include: ‘Better than usual’, ‘Same as usual’, ‘Less than usual’, ‘Much less than usual’. The scale measures transitory distress. A scoring key of 0–3 was used to determine totals for the analysis and a scoring key of 0, 0, 1, 1 was used to determine ‘caseness’ or those ‘at risk’, where totals on the measure above 3 indicated a risk of developing a stress-related illness [50]. The Alpha coefficient was .89.

#### Hospital anxiety and depression scale (HADS) [56]

The anxiety sub-scale of the HADS was used to measure anxiety. Respondents rated seven statements, each on a scale from 0–3, where 0 is “not at all” and 3 is “most of the time”. An example item is: “I feel tense or wound up”. The Alpha coefficient was .87.

#### The course satisfaction scale (abridged from the national student survey, [52]

This is a three-item scale, using a 5-point Likert scale. Respondents are asked to rate statements that describe their course, such as: ‘I enjoy my studies.’ The Alpha coefficient was .89.

#### Ethics

The study received ethical approval from the Ethics committee at the host university. All participants received a brief and a point of contact for further clarifications. All were informed that participation was voluntary and they were free to stop at any time and all acknowledged informed consent before participating, in accordance with the Declaration of Helsinki.

## Results

One sample *t*-tests were carried out to compare the mental health of the students studying during the pandemic compared to pre-pandemic normative data (H1). Independent sample *t*-tests were carried out between those ‘at risk’ and ‘not at risk’ on sources of stress (H2) and between those identified as high in defensive pessimism and optimism on learning motivation (H5). Multiple hierarchical regressions were run using SPSS version 27. Predictors were entered in line with the Transactional model—Sources of stress (primary appraisal factors) were entered in block one and personality and the influences on coping (secondary appraisal factors) in block two, along with demographics. The regression tables illustrate the final block for each model. Regression assumptions were checked and confirmed, and the guidelines proposed by Baron and Kenny (1986) were followed to arrive at the most parsimonious model and in testing for moderation [57].

The GHQ results in this sample (M = 18.44, SD = 7.40) were compared with and significantly higher than normative data from James, Yates and Ferguson (2013) with a cohort (n = 251) of UK (medical) students (M = 13.39, SD = 5.77), *t*(159) = 8.63, *p* < .001 [58]. The scores were computed for caseness and 68.5% (n = 111) were ‘at risk’ and 30.2% (n = 49) ‘not at risk’. This compares with 19% ‘at risk’ in the Health Survey for England report (n = 8034) [19]. This supports H1.

Table 1 compares those ‘at risk’ and ‘not at risk’ on stress ratings for the different sources of stress from the NSS scale, for example, those ‘at risk’ more frequently perceived ‘Teaching on my course’ (one of the demands they were asked to rate) as a hassle, compared to those ‘not at risk’:

**Table 1:** *t*-test results comparing those ‘at risk’ and ‘not at risk’ on ratings of NSS stress demands

Group	Mean	SD	T value
‘At risk’ Teaching on my course Hassle	3.85	2.26	3.08**
‘Not at risk’ Teaching on my course Hassle	2.75	1.54	
‘At risk’ Teaching on my course Uplift	6.46	1.71	1.16
‘Not at risk’ Teaching on my course Uplift	6.65	2.06	
‘At risk’ Time management Hassle	7.80	1.64	4.66***
‘Not at risk’ Time management Hassle	6.45	1.71	
‘At risk’ Time management Uplift	4.60	2.38	1.68
‘Not at risk’ Time management Uplift	5.29	2.41	
‘At risk’ Intellectual stimulation Hassle	4.78	2.10	1.95*
‘Not at risk’ Intellectual stimulation Hassle	4.10	1.87	
‘At risk’ Intellectual stimulation Uplift	6.55	1.99	1.16
‘Not at risk’ Intellectual stimulation Uplift	6.96	2.27	
‘At risk’ Peer support Hassle	3.32	2.70	2.87**
‘Not at risk’ Peer support Hassle	2.08	1.93	
‘At risk’ Peer support Uplift	7.17	2.54	1.55
‘Not at risk’ Peer support Uplift	7.83	2.33	
‘At risk’ Tutor support Hassle	3.13	2.54	2.48**
‘Not at risk’ Tutor support Hassle	2.06	2.28	
‘At risk’ Tutor support Uplift	6.60	2.92	1.44
‘Not at risk’ Tutor support Uplift	7.30	2.63	
‘At risk’ Wider university support Hassle	4.02	2.60	1.97*
‘Not at risk’ Wider university support Hassle	3.15	2.52	
‘At risk’ Wider university support Uplift	5.60	2.48	0.51
‘Not at risk’ Wider university support Uplift	5.82	2.54	
‘At risk’ Family and friends support Hassle	2.61	2.52	3.68***
‘Not at risk’ Family and friends support Hassle	1.15	1.60	
‘At risk’ Family and friends support Uplift	7.91	2.45	1.76
‘Not at risk’ Family and friends support Uplift	8.67	1.94	
‘At risk’ Social opportunities Hassle	4.85	2.86	1.21
‘Not at risk’ Social opportunities Hassle	4.23	2.98	
‘At risk’ Social opportunities Uplift	4.35	2.81	1.76
‘Not at risk’ Social opportunities Uplift	5.22	2.76	
‘At risk’ Assessment Hassle	2.76	1.41	1.83
‘Not at risk’ Assessment Hassle	2.31	1.56	
‘At risk’ Assessment Uplift	2.85	1.39	0.19
‘Not at risk’ Assessment Uplift	2.90	1.46	
‘At risk’ Workload Hassle	8.15	1.79	3.91***
‘Not at risk’ Workload Hassle	6.61	2.47	
‘At risk’ Workload Uplift	4.45	2.70	1.62
‘Not at risk’ Workload Uplift	5.18	2.46	
‘At risk’ Partner Hassle	3.54	2.40	3.73***
‘Not at risk’ Partner Hassle	2.00	2.19	
‘At risk’ Partner Uplift	6.81	2.58	0.481
‘Not at risk’ Partner Uplift	7.04	2.80	

*p < .05; **p < .01; ***p < .001

There were significant differences in nine out of eleven stress demands, when rated as a hassle, with those ‘at risk’ rating the demands higher than those ‘not at risk’. There were no significant differences in the uplifting ratings between these two groups. This supports H2 for the difference in hassle ratings for the two groups, but not for the difference in uplifting ratings.

### Pandemic-related stressors

Those ‘at risk’ (n = 110), (M = 15.55, SD = 4.01), compared to those ‘not at risk’ (n = 49), (M = 12.93, SD = 4.98), spent more time on their devices, *t*(157) = 3.51, *p* = .001, and the ‘at risk’ group (n = 110), (M = 16.79, SD = 3.58), compared to those ‘not at risk’ (n = 49), (M = 11.08, SD = 4.80), scored higher on lack of motivation, *t*(157) = 8.32, *p* < .001. This supports H2.

The regression model explained, 63.4% of the variance in scores on GHQ (Table 2). The results of the regression indicated that there was a collective significant effect between lack of motivation, neuroticism, context control, optimism and openness on mental health, *F*(6, 145) = 44.54, *p* < .001, R2 = .648, Adjusted R2 = .634). The individual predictors were examined further and indicated that: Lack of Motivation, Beta = .33, (*p* < .001); neuroticism, Beta = .28, (*p* < .001); context control, Beta = − .26, (*p* < .001); and optimism, Beta = − .14, (*p* = .024) and openness, Beta = .10, (*p* = .041) were predictors in the model and offered partial support for H3.

**Table 2:** Multiple regression with GHQ: regressed on the cluster of uplifting stressors, pandemic-related stressors and on personality and control

Model	Unstandardised Coefficients		Standardised Coefficients
	B	Std. Error	Beta
(Constant)	26.06	4.16	
Teaching on my	− .42	.19	− .11*
Course uplift			
Lack of motivation	− .51	.09	.33***
Context control	-1.00	.23	− .26***
Optimism	− .33	.14	− .14*
Neuroticism	1.07	.25	.28***
Openness	.55	.27	.10*

*p < .05; ***p < .001

### Optimism and pessimism as predictors of learning motivation

Following the procedure first adopted by Norem and Cantor (1986), the participants in the upper quartile on defensive pessimism were identified and, from this group, those in the upper quartile on anxiety and course satisfaction were selected and they were compared with those in the upper quartile on optimism. Results of the independent sample *t*-tests indicated no significant differences in learning motivation between the 11 participants selected for being in the upper quartile on defensive pessimism, anxiety and course satisfaction (M = 7.6, SD = 1.2) compared with the 44 participants in the upper quartile on optimism (M = 7.2, SD = 2.1), (*t*(53) = .56, *p* = .159). There were no significant differences between the defensive pessimism group (M = 12.86, SD = 2.07) and the optimism group (M = 12.83, SD = 2.50) on course satisfaction (*t*(53) = .04, *p* = 967), with the defensive pessimism group scoring higher (M = 18.55, SD = 2.50) than the optimism group (M = 8.45, SD = 4.83) on anxiety (*t*(53) = 6.68, *p* < .001). This supports H5 – defensive pessimism was just as effective as optimism on learning motivation for those anxious-prone.

However, significant differences were reported between the defensive pessimism group (M = 3.6, SD = 1.5) and the optimistic group (M = 5.1, SD = .9) on satisfaction in life, (*t*(53) = 3.97, *p* < .05) and between the defensive pessimism group (M = 10.6, SD = 4.5) and the optimistic group (M = 16.9, SD = 2.4) on happiness, (*t*(53) = 6.40, *p* < .05).

The optimistic group scored higher on context control (M = 6.94, SD = 1.75) than the defensive pessimism group (M = 4.45, SD = 1.06), (*t*(53) = 4.49, *p* < .001). However, there was no evidence that context control played a mediating role between optimism and life satisfaction or between optimism and happiness.

The regression model explained 40.9% of the variance in scores on learning motivation (Table 3). The results of the regression indicated that there was a collective significant effect between teaching demands rated as hassle, social opportunities rated as an uplift, lack of motivation, conscientiousness and teaching on my course hassle-conscientiousness moderator on learning motivation, *F*(5, 144) = 21.59, *p* < .001, R2 = .43, Adjusted R2 = .41). The individual predictors were examined further and indicated that: teaching demands rated as hassle, Beta = − .37 (*p* < .0001); social opportunities rated as an uplift, Beta = .22 (*p* < .001); lack of motivation, Beta = − .22 (*p* < .001); and conscientiousness and teaching on my course hassle-conscientiousness moderator, Beta = .13 (*p* < .05) were predictors in the model and offered partial support for H3.

**Table 3:** Multiple regression with learning motivation: regressed on the cluster of ‘hassle’ and ‘uplifting’ stressors, pandemic-related stressors and on personality

Model	Unstandardised Coefficients		Standardised Coefficients
	B	Std. Error	Beta
(Constant)	6.98	.75	
Teaching on my	− .32	.06	− .37***
course hassle			
Social opportunities uplift	.14	.04	.22***
Lack of motivation	− .08	.02	− .21***
Conscientiousness	.28	.08	.24***
Teaching on my course hassle- Conscientiousness moderator	.07	.04	.16*

*p < .05; ***p < .001

**Fig. 2:**
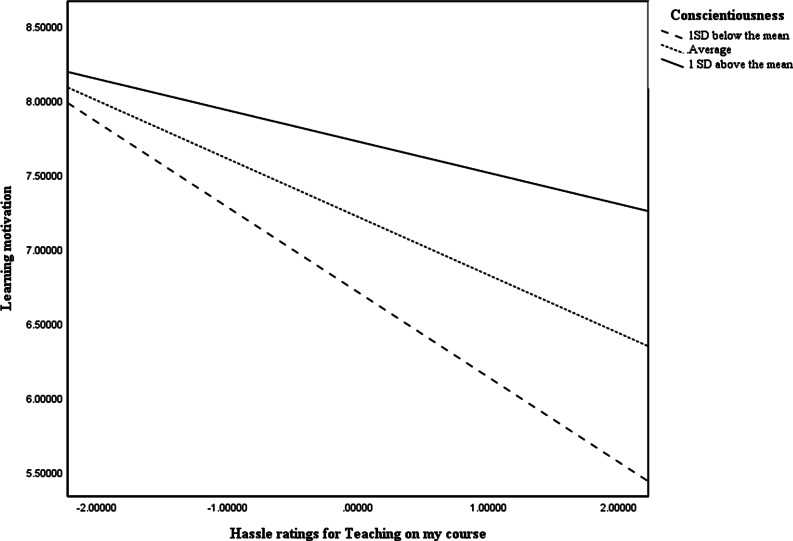
Slope graph testing the interaction between conscientiousness and teaching demands rated as hassle on learning motivation

The results indicate that high levels of conscientiousness moderated the effects of teaching demands students found disruptive during a pandemic on learning motivation (Fig. 2). This supports H4.

## Discussion

### The ‘at risk’ caseness analysis

A striking finding, related to H1, was that 68.5% of respondents were ‘at risk’ of a stress-related illness. This exceeded that reported in pre-pandemic populations of students and non-students of similar age [13, 19, 20] and the average GHQ score was higher compared with pre-pandemic normative student populations [58]. This reflects the marked stress associated with living and learning during a pandemic and the pattern of results is consistent with that found across Europe and the international comparisons made earlier [27–33].

For H2, the NSS items were sub-divided into eleven factors or stress demands, rated once as a hassle and again as an uplift. For nine of eleven of these factors, those ‘at risk’ scored higher on hassles ratings compared with those ‘not at risk’ (Table 1). Some of these differences may, in part, be attributable to actual differences in the demands. For example, there may be differences in the quality of support offered between peers or from tutor to tutor or from one’s family and friends.

However, for other demands, such as the teaching experience, workload and course resources (a source of intellectual stimulation), these were the same or similar. That is, students followed the same modules, received the same pre-recorded lectures and faced the same assignments. So, the differences in hassles ratings for these demands was more likely to be attributable to differences in student appraisals, with those ‘at risk’ and, by implication, not coping well, more likely to interpret those demands as distressing. It is possible that some of those ‘at risk’ were not, de facto, bad at coping but given these different appraisals to the same stressors, the contention offered here is that *most* in the ‘at risk’ group could improve in how they cope. That it was differences in the individual coping rather than material differences in the stressors faced, is supported by the finding in relation to pandemic-related stress: Those ‘at risk’ spent more time on their devices and they were more likely to struggle to find the motivation to be productive and they more often reported changes in sleeping habits i.e., they engaged in behaviours that impacted on their coping or reflected poor coping.

### The mental health regression analysis

For H3, in this analysis (Table 2), lack of motivation was the strongest predictor of adverse mental health and it referred to the loss of mojo or motivation towards learning demands during a pandemic. It appears that apathy and a lack of energy to undertake necessary tasks was a major source of stress. Procrastination is a perennial problem for most people from time to time and frequently for students. It is a state that is negatively reinforcing but avoidance adversely impacts on learning and wellbeing [13, 59]. The challenge of studying during a pandemic has created a set of circumstances where, despite one’s aspirations, struggling to overcome a state of procrastination proved especially difficult and this was the strongest predictor of adverse mental health.

Those students that are worry-prone or anxious by nature appear to suffer most. This was suggested by the positive correlation between neuroticism and GHQ. Consistent with earlier research [13, 41], developing a sense of control in specific contexts is a powerful coping mechanism – high scores on context control were associated with low scores on GHQ.

Optimism was predictive in the same way but weaker than context control. Several studies lay testament to the value of optimism, but others show that it either does not feature or is only weakly related to measures of wellbeing [13, 38]. Context control is a frequent robust predictor of health and wellbeing and this measure of control is learnt, not dispositional [40]. Developing context control holds potential to help students cope in the face of changing pandemic challenges. Both context control and optimistic strategies can be developed through learnt strategies, however, based on these findings, it is the former that may offer more benefits.

Openness was a significant predictor. Higher scores on openness were associated with higher scores on GHQ. On the face of it, this suggests that openness has an adverse effect on mental health. However, it is important to remember that stress is not always distress and if one is to learn and develop new knowledge and new skills, one has to be willing to move out of one’s comfort zone. Feeling vulnerable and accepting that one might get things wrong and make mistakes and accepting that one’s self-esteem may take a hit in some disappointing marks or critical feedback, for example, are best interpreted as the growing pains of a growth mindset [60]. Hand-in-hand with this, is the feeling that one may occasionally doubt that one can meet the learning challenge. This is consistent with the large number of students who eventually succeed, if not shine, in their performance but who experience imposter syndrome en route [61]. Being open-minded is integral if one is to effectively master new learning, and so too is its association with heightened stress.

‘Teaching on my course’, when rated as an uplift, was associated with lower scores on GHQ. This is likely to reflect the efforts by faculty to engage their students remotely and to provide effective teaching through pre-recordings, live seminars and more frequent live tutorials (these were held weekly instead of fortnightly, the pre-pandemic format). It also reflects the tendency by those scoring high on optimism, to more readily interpret stress demands as opportunities to achieve.

Openness and high scores on idealism, as opposed to cynicism, have been associated with more frequent and more intense experiences of ‘elevation’. This is an uplifting emotion, where one feels inspired, experiences awe or a general feeling of emotional warmth [62]. Teaching and learning experiences are more likely to be elevating if one adopts an open-minded perspective and this might be part of the explanation behind the dominance of the uplifting ratings for teaching and the openness predictor in the model.

Support, as a coping resource, was removed in the process of arriving at the most parsimonious regression model. This is not to suggest that support is not important. The literature supporting its efficacy is strong [37]. Its absence here could be attributed to the lack of in-person support during the pandemic and its significance was over-shadowed by the importance of those predictors in the model. In the second analysis it did feature, in the form of social opportunities. This suggests that it remains important, but less so in predicting mental health, as measured by the GHQ.

### Is there a place for defensive pessimism in coping?

For H5, defensive pessimism was helpful for those anxious-prone in relation to learning motivation: There was no difference in levels of learning motivation between those respondents high on defensive pessimism and anxiety compared with those high on optimism. This suggests that for individuals who are anxious-prone, rather than adopt those ubiquitous optimistic thinking strategies, setting unrealistically low learning expectations, might relieve them of the pressure to achieve and actually (ironically) enhance performance. Only those in the upper quartile on defensive pessimism, anxiety and course satisfaction were selected and compared against those in the upper quartile on optimism. Selecting those high in course satisfaction was used because it made it more likely that their pessimism was defensive not realistic – the satisfaction rating was an indicator that they had been achieving. Had those with lower scores in course satisfaction been included it would make it more likely that their pessimism was, for some, a realistic reflection of a disappointing course performance.

### A cautionary note

It was noteworthy that while not hypothesized, defensive pessimism did not offer the same dividends for happiness and satisfaction with life. The defensive pessimist group scored significantly lower than the optimists on these measures. Moreover, optimism remained a significant predictor of happiness and life satisfaction when context control was tested as a potential mediator. This suggests that for anxious-prone individuals, defensive pessimism offers an effective strategy for harnessing motivation towards learning goals, but optimistic thinking strategies *and* context control should be employed to help bolster these other wellbeing ingredients.

Consistent with the tenets of positive psychology, one does not always need to work directly on one’s coping deficits, such as trying to lower measures of neuroticism. Rather, if one focuses on building one’s coping strengths, such as improved techniques in context control and in optimistic thinking strategies, and in defensive pessimism for those anxious-prone, it can buffer against the costs of neuroticism on mental health [47, 48].

### Regression analysis for learning motivation

For H3, in this analysis (Table 3), teaching demands was the strongest predictor of learning motivation but not in the positive way observed in the first regression – the more these demands were rated as a hassle, the more learning motivation declined. The benefit of asking participants to identify the distress and eustress elements of demands allows one to identify their subtle and disparate influences. The nature of learning and teaching took on a new meaning when students did it virtually and in isolation, and in a way that involved many more hours sat in front of a computer screen. Where faculty introduced changes that helped, it significantly improved mental health (Table 2). However, so dramatic were the changes in learning that this inevitable shift in practice is likely to be associated with added hassle ratings. If there are other added disappointments, perhaps related to teaching variability or in the levels of effort faculty engaged in to support students, then it is understandable that these combined influences had an adverse impact on learning motivation.

Previous research justified testing the role of personality, support and control but several key influences—extraversion, neuroticism, control and optimism, did not feature in this second analysis. Conscientiousness did however, and it was the most effective in maintaining learning motivation. It is likely that the isolation of the pandemic meant there was little scope to derive the same wellbeing benefits (for example in happiness and general motivation) that extraversion is normally associated with [63]. Studying remotely and virtually put an increased importance on how learning and teaching was delivered and rated and, not unsurprisingly, when the experience was positive it was rated very favourably (its uplifting rating in the first regression) and when it was disappointing, it had a greater adverse impact on learning motivation because the pandemic-induced isolation took away most of the coping benefits that come from being extraverted.

The items underpinning the social opportunities predictor asked respondents to rate opportunities to interact with other students on the course and in university clubs and societies. The predictor represents a proxy for support. Its positive relationship with learning motivation shows that, despite the restricted opportunities imposed by the pandemic, having the contact and support of other students, whether course-related or recreationally, increased learning motivation.

Consistent with the mental health regression, students who reported losing general motivation as a fall-out of the prolonged Covid restrictions, found this carried over to the motivation towards their studies. In both regressions, as part of H4, all the predictors were tested for moderation effects and the slope graph in Fig. 2 illustrates the moderating influence of conscientiousness on learning motivation in response to teaching demands: For those low in conscientiousness (the bottom line), the more teaching demands were experienced as a hassle the more dramatically learning motivation declined. For those average in conscientiousness (the middle line) the decline in learning motivation was less dramatic. For those high in conscientiousness (the top line), motivation was higher and increased ratings of teaching as a hassle had only a nominal influence on rates of learning motivation compared to the other two groups. This suggests conscientiousness was an important buffer for learning motivation against the adverse changes in the quality of teaching.

## Limitations

The NSS was used because it is recognized as the, de facto, measure of student experience. However, the evidence of its validity does not yet match the frequency of its use [64]. The use of a survey method and volunteer sample are not without limitations and while the sample size was good, relative to target population, a larger sample across all cohorts in the psychology department would have allowed more insights into the difference demands faced in each year of study.

Norem and Cantor (1986) used upper quartile measures on GPA to benchmark those respondents whose pessimism was likely to be defensive not realistic. Here, course satisfaction was used. While past performance is likely to be an influence on course satisfaction, it is not the only influence – so is the quality of teaching and how engaging learning resources might be. This may question the validity of using course satisfaction alone to identify those that are defensive rather than realistic pessimists. Using course satisfaction *and* GPA, rather than either alone, would be a useful way to increase the confidence in identifying those whose pessimism was defensive.

Identifying the sources and experience of stress that are likely to enhance performance and are thereby uplifting as opposed to a hinderance or hassle, is a key challenge for those of us who explore this aspect of positive psychology. The stress that helps you achieve may be experienced as unpleasant and unwanted at the time and, because of that, be more likely to be rated as a hassle. This was the argument offered to explain the relationship between openness and GHQ. A fuller explanation on the distinction between the sources of stress that can help and that can inhibit performance was added to the participant brief in this study, compared to similar earlier studies, but, as an online survey, it was difficult to drive home this distinction. An improvement might be to adopt different labels for ‘hassles’ and ‘uplifts’ such as sources of stress that ‘hinder’ performance and that are ‘necessary to facilitate’ performance.

Overall, the lack of motivation was acute. While positive ratings of teaching and optimistic thinking were associated with good mental health, context control was a stronger predictor. Support and conscientiousness were positively associated with learning motivation, and conscientiousness buffered against the adverse impact of stress on motivation. Openness was associated with the stress involved in learning and, for those anxious-prone, defensive-pessimism was as effective as optimism in stimulating learning motivation.

## Recommendations

Studying during a pandemic imposed dramatic and significant changes in student learning and coping. The interpretation offered suggests specific pointers to help students cope; to improve mental health and learning motivation. During induction and early in their studies, students could be offered resilience training that includes tips on the thinking strategies adopted by optimists (for example, that change can be construed as a challenge even if one’s initial reaction is one of threat; in defensive optimism, active disputing, problem-based coping) and in defensive pessimism for those high in anxiety or who experience situations associated with high anxiety, such as early in the semester for new and returning students. It would be useful to raise awareness to re-interpret ‘stress and change’ in a positive light. Understanding our evolved tendency to perceive change as a threat is, to that end, likely to improve coping.

Control, in an education context, could be developed by empowering students with an HE skill-set that goes beyond exercises in time and task management, important though they are, and that incorporates apps that imbed daily and weekly schedules anchored around assignment deadlines; for better time management, and that utilize evidence-based positive psychology techniques. Students can be supported in their learning independence by using some of the psychology-based apps designed for this purpose; along with selected subject-specific podcasts to help enthuse them in their learning and to help move them from a lay understanding to a progressively more academic and in-depth understanding at a pace that leaves them feeling in control.

As universities move to return to in-person teaching, they are more likely to retain some elements of virtual learning. Both regression analyses showed this can be associated with uplifting *and* hassle ratings. It is important, therefore, to look to maximise its positive impact. For example, by recording virtual learning for students to revisit; using transcript options to facilitate (not replace) student note-taking; allowing student participation through chat features and break-out rooms. Many faculty drew on these elements and are getting better at doing this. However, during this study, there was a mixed take-up in encouraging students to turn on their cameras during learning and where some educators did not turn on their camera when presenting. Evidence in multi-sensory processing [65] and the animacy effect in memory [66], support the benefit to learners if they can see as well as hear each other and the presenter. Finally, support opportunities should continue to be enhanced through extended freshers’ fayre events; student inductions with a strong peer-networking focus, along with peer-mentoring initiatives.

These are just some suggestions to help develop specific personality ingredients; student control and support and which, in turn, increases the likelihood that a conscientious approach is one that quickly translates into effective learning and coping. These initiatives hold the potential to combat procrastination, improve learning motivation and mental health.

## Data Availability

The data set is available at: https://orcid.org/0000-0001-6631-721X. The question items are subject to copyright but the sources for all the measures used are referenced and interested parties can contact any of these sources. The authors vary on their decisions to make their tests available for free for educational purposes.
